# Experimental study of asphaltene deposition during CO_2_ and flue gas injection EOR methods employing a long core

**DOI:** 10.1038/s41598-024-54395-0

**Published:** 2024-02-14

**Authors:** Mehrdad Jalili Darbandi Sofla, Zohreh Dermanaki Farahani, Salman Ghorbanizadeh, Hamed Namdar

**Affiliations:** 1 Special Core Analysis Laboratory, Petro Vision Pasargad Oil and Gas Company, Tehran, Iran; 2 Department of Petroleum Engineering, Faculty of Chemical Engineering, Tarbiat Modares University, Tehran, Iran

**Keywords:** Permeability impairment, Asphaltene deposition, Enhanced oil recovery, Core flooding, CO_2_ injection, Flue gas, Geochemistry, Geodynamics, Petrology

## Abstract

Gas injection is a well-known method for enhancing oil recovery (EOR). The utilization of greenhouse gases, such as carbon dioxide (CO_2_) or flue gas, offers the dual advantage of reducing greenhouse gas emissions while potentially enhancing the sweep efficiency in oil recovery. Nevertheless, one of the notable challenges encountered when using these gases is the precipitation and deposition of asphaltenes, leading to formation damage and a decrease in reservoir permeability, particularly in the case of light oil reservoirs. In this study, CO_2_ and flue gas were injected into an elongated core sample comprising four individual core plugs under reservoir conditions to displace the light live oil. The recovery factor and asphaltene deposition along the core holder were assessed and compared as two crucial parameters within the gas injection scenario. Our results indicate a significantly higher recovery factor of 86% achieved with CO_2_ injection compared to 36% with flue gas injection, attributable to differences in their interfacial tension and miscibility. However, the CO_2_ injection method exhibits more pronounced formation damage. Individual assessment of each core plug reveals that permeability impairment is most acute in the initial two core plugs, situated closer to the injection face of the extended core. These findings enhance our understanding of the mechanisms contributing to permeability impairment resulting from asphaltene deposition during CO_2_ and flue gas injection for EOR.

## Introduction

With global population growth and industrialization, the need for energy has increased rapidly throughout time^1,2^. Although much progress has been made, renewable energy schemes suffer from high costs, difficulties in execution, and unreliability. Additionally, they are unable to supply the planet with a substantial amount of energy^3^. Crude oil, on the other hand, is the world's most important energy source and will remain so in the future^4^. In addition, typical recovery procedures, with the residual oil saturation around 60%, lack the efficiency required for effective oil extraction^5^. In this regard, various successful enhanced oil recovery (EOR) strategies have been established, which research and development are of great importance^6^. The three major EOR procedures are gas injection, thermal injection, and chemical injection recovery^7^. EOR techniques that involve gas flooding are widely used in the oil industry and are among the most reliable^8,9^.

Even though a variety of gases are employed for gas injection, CO2, natural gas, and nitrogen are regarded among the most promising ones^10^. Due to its high sweep efficiency and successful function in reducing greenhouse gas emissions, CO_2_ injection has garnered a lot of interest since 1950^11,12^. In order to examine the effectiveness of the CO_2_ injection process, several pilot tests were carried out. Some of them proved to be a big success, recovering 70% of the oil that was initially present^13^. Moreover, CO_2_ can easily reach the supercritical state, where its density is nearly identical to that of a liquid and its viscosity is comparable to that of a gas. Also, it has higher injectivity than water due to its low viscosity. The diffusion coefficient of CO_2_ is up to 100 times that of liquids due to its extreme solubility in oil^14–17^. Flue gas has also aroused a lot of attention as an EOR agent in recent years^18^. It is nearly free, simple to get, and its injection, similar to the injection of CO_2_, helps to reduce greenhouse gas emissions. Therefore, many researchers and companies are evaluating its injection as an EOR approach with high potentials^19^.

Although the recovery factor increases when these methods are employed, the possibility of asphaltene precipitation poses a severe technical obstacle to these advantageous EOR approaches^20^. One of crude oil's four main constituents is asphaltene. Resins, aromatics, and saturates form the other three. Asphaltenes and resins are the crude oil’s polar fractions^21^.

Resins play a critical role in ensuring that asphaltene particles in crude oil remain stable. Since the resins have both polar and nonpolar sites, they can surround asphaltene particles and act as a bridge between the nonpolar component of the crude oil and the polar asphaltene particles^22,23^. When the resin concentration is inadequate, the asphaltene particles come together to form a big molecular group because the surrounding layer cannot be created or is too thin to stabilize the crude oils asphaltene particles effectively^24^. That is why, despite their larger asphaltene concentration, heavy oils have a lower tendency to precipitate due to a large amount of resin available to stabilize the asphaltene particles. Therefore, asphaltene precipitation problems are more common in reservoirs with light to medium crude oil and low asphaltene concentration^25,26^.

Until the reservoir conditions change (such as temperature, crude oil composition, or pressure) the collaboration between asphaltenes and resins will remain in thermodynamic equilibrium^27^. During the injection process, the CO_2_ gas dissolves in the oil and mobilizes the crude oil's lighter components^28^. As a result, the composition of crude oil will drastically change and disrupt the existed thermodynamic equilibrium^29,30^. The onset of precipitation of the asphaltene particles happens at this point, meaning they solidify and form a new solid phase in the solution^31^. The precipitated asphaltenes either suspend in the crude oil and flow away, or deposit on the porous media surface^32^. As soon as the asphaltene particles settle on the pore surface, they may cause formation damage by narrowing the pore throats^33,34^. By attaching to the rock surface, they can alter the oil-wetness of reservoir rock, as a result, the oils effective permeability decreases and its irreducible saturation increases. and as a result declining the performance of the process^35,36^.

Concerns about asphaltene deposition present a significant challenge to flow assurance, and among other factors, it is primarily responsible for crude mobility resistance^37,38^. The petroleum industry has been concentrating on preventing this issue since it is one of the most severe and expensive technical difficulties^39^. Thus, prior investigations are critical in the fruitful implementation of a gas injection. CO_2_ and flue gas injection EOR procedures as well as the associated risk of asphaltene deposition during these operations have been extensively studied. Zanganeh et al. (2012) monitored asphaltene deposition in CO_2_ miscible gas injection process under various condition on a rock by high quality microscopic image. Then these images were processed to calculate the amount of asphaltene deposition and its size distribution. Results indicated that asphaltene deposition reduced by pressure depletion. Also, with the concentration of injected CO_2_, the confirmation of increased asphaltene deposition is evident in the results of CO_2_ gas injection. In addition, they mentioned that the molecular structure of asphaltene might exert a discernible impact on the deposition of asphaltene. An increase in temperature from 35 to 90 °C fosters the growth and aggregation of asphaltene particles^39^. Soroush et al. (2014) showed that the extent of pore plugging damage will be significantly diminished below the minimum miscibility pressure (MMP) for CO_2_ in comparison to its effect above the MMP^40^. In order to understand asphaltene deposition in low permeability reservoirs during CO_2_ flooding, a simulation study was carried out by Mohammed et al. (2017). Their suggestion involved optimizing CO_2_ injection through the use of cyclic CO_2_-brine flooding^41^. Wang et al. (2018) compared the effect of nitrogen and carbon dioxide injection on the pressure of asphaltene deposition with the help of flash separation tests and simulation using PVTsim Nova software. They found that the amount of light components up to C_4_ and heavy components C_36+_ reduces with the increase of nitrogen gas and carbon dioxide injection volume. But carbon dioxide has more extraction. Also, in the injection of nitrogen gas, the pressure of the asphaltene deposition is higher and it creates less problems in the deposition of asphaltene compared to the carbon dioxide gas^42^. In an experimental study, Fakher et al. (2020) investigated the effect of factors that affecting asphaltene stability during the injection of CO_2_ in order to reduce the problems caused by asphaltene deposition. They investigated the effect of CO_2_ gas injection pressure, oil viscosity and pore size, and the results were correlated with the Yen-Mullins asphaltene model. The results showed that asphaltene concentration increases with increasing viscosity, and also with decreasing pore size, filter cake thickness increases. Asphaltene filter cake increases because by reducing the pore size, the smaller pores are plugged by asphaltene particles, which makes larger asphaltenes unable to pass through^43^. Parsaei et al. (2020) investigated the effect of nanoparticle on asphaltene precipitation during CO_2_ injection by using vanishing interfacial tension technique. Results shown that the presence of iron oxide nanoparticles can reduce asphaltene precipitation. Also, minimum miscibility pressure decreased by addition of nanoparticle^44^. Wang et al. (2020) investigated the damages caused by asphaltene precipitation during the injection of water alternating flue gas (WAFG). They concluded that although WAFG injection after water injection can increase production efficiency, asphaltene precipitation has significant effects on petrophysical properties and should be investigated. Also, their results showed that the lower quality of the petrophysical properties, more precipitation will be retained and have a greater effect. That's why the effect of sediment on conglomerate is more intense than on sandstone^45^. Xiong et al. (2023) studied the effect of methane, carbon dioxide and nitrogen injection on asphaltene deposition. The results showed that asphaltene precipitation is related to the miscibility of gases. Similar to previous studies, they also stated that nitrogen gas injection causes less asphaltene deposition. Also, carbon dioxide gas causes more asphaltene deposition than methane gas^46^. Gandomkar et al. (2023) investigated the reduction of asphaltene precipitation by modifying carbon dioxide gas with toluene. They concluded that the injection of toluene-carbon dioxide solution caused a decrease of 3.7% and 0.7% of asphaltene precipitation in two samples of asphaltenic oil^47^. In a laboratory study, Amraeiniya et al. (2023) studied the prevention of asphaltene precipitation during the injection of carbon dioxide gas with the help of Al_2_O_3_ nanoparticles at various pressures and at a temperature of 60 °C. They stated that Al_2_O_3_ nanoparticles have a better effect in reducing asphaltene precipitation in oil with a lower higher H/C ratio and higher nitrogen component^48^.

However, these studies have rarely been dynamically examined under conditions typically found in a reservoir using subsurface sampled live oil and reservoir rock samples arranged as a long core. The objective of this work is to perform a detailed study of the potentials and risks of CO_2_ flooding EOR in comparison to the less expensive and more easily accessible flue gas in one of the Iranian reservoirs (in the south of Iran). Moreover, the intensity of formation damage and the amount of the deposited asphaltene are evaluated in different parts of the long core representing various parts of the reservoir around the injection well. To displace the live oil, the gas sample (CO_2_ or flue gas) was injected into the long core composed of 4 core plugs. The reason for choosing four separate standard short core plugs instead of a single long core plug was to allow us to focus on the permeability impairment and the amount of asphaltene deposition in different sections of the assembled long core without having to cut it into sections and damage its porous medium structure. The outcomes of this investigation will assist us in developing a better grasp of the underlying reasons behind permeability reduction caused by asphaltene deposition during CO_2_ and flue gas injection EOR methods.

## Experimental section

### Materials

#### Live oil

Subsurface sampling procedure (API-RP-44) was employed to collect live oil samples. Saturate, aromatic, resin and asphaltene fractions of the oil (SARA fraction analysis) are presented in Table 1. Table 2 lists additional oil specifications as well as reservoir specifications.

**Table 1:** The results of SARA analysis.

Type of group	Weight percent in the live oil
Saturate	47.92
Aromatic	37.02
Resin	14.95
Asphaltene	0.11

**Table 2:** Reservoir and oil specifications.

Reservoir depth	3900 (m)
Reservoir initial pressure	6500 (psi)
Reservoir temperature	112 (°C)
Reservoir saturation pressure	4394 (psi)
Solution gas/oil ratio (GOR)	1113 (SCF/STB)
Oil formation volume factor at reservoir temperature and initial pressure	1.5975 (bbl/STB)
Density of oil at reservoir temperature and initial pressure	0.6931 (g/cm^3^)
API gravity of residual oil	32.4
Specific gravity of residual oil @ 60/60 $^\circ{\rm F}$	0.8633
Molecular weight of the residual oil	243 (g/mol)
Viscosity of the oil at reservoir temperature and initial pressure	1.7124 (cp)
Molecular weight of the reservoir oil	94 (g/mol)
Molecular weight of C_12+_	498
Specific gravity of C_12+_ @ 60/60 $^\circ{\rm F}$	0.9233
Live oil-CO_2_ IFT at reservoir pressure and temprature	0.43 dyn/cm
Live oil-flue gas IFT at reservoir pressure and temprature	4.58 dyn/cm

#### Reservoir brine

Table 3 displays the ionic composition of the reservoir brine. Brines density and viscosity were 1.644 cP and 1.146 g/cm^3^, respectively.

**Table 3:** The ionic composition of reservoir brine.

Ion	${{\text{Na}}}^{+}$	${{\text{Ca}}}^{2+}$	${{\text{Mg}}}^{2+}$	${{\text{Cl}}}^{-}$	${{\text{SO}}}_{4}^{2-}$	TDS
Concentration (mg/l)	54,918	8,960	826	103,305	480	168,489

#### Synthetic oil

In order to centrifuge the rock samples to reach irreducible water saturation, synthetic oil was prepared by combining the proper amount of kerosene and liquid paraffin (Merck, Germany). Its viscosity and density were 6.3 cP and 0.81 gr/cm^3^, respectively. The generated synthetic oil and brine had the same viscosity ratio as live oil and brine in the reservoir conditions.

#### Core plugs preparation and long core assembly

A total of 44 core plugs was cut from whole-core plugs of the southwestern Iranian reservoirs. The core plugs were then washed using a two-stage cleaning method including Soxhlet extractors. In the first and second stages of cleaning, toluene (Merck, Germany) and subsequently methanol (Merck, Germany) were used to remove organic materials and inorganic salts, respectively. To check that if the procedure was complete, the examination of the effluent was performed repeatedly until it was clear under UV light in the first stage, and silver nitrate titration showed that it was free of any inorganic salts in the second stage.

Routine core analysis, including Helium porosity and steady-state gas permeability measurements, were performed after drying the core plugs in an oven. Three criteria were defined to choose four of them for further experiments. The first criterion was the similarity of petrophysical properties. The second one was based on the results of their CT scan analysis, which showed that they had no joints or fractures and were homogeneous enough to be considered suitable for flooding experiments. As the third criterion, the liquid permeability of the core plugs was between 1 and 2 mD to provide a better representation of the reservoir's overall characteristics. Table 4 lists the petrophysical characteristics of the core plugs.

**Table 4:** The petrophysical characteristics of the core plugs.

Plug ID	Diameter (cm)	Length (cm)	Dry weight (gr)	Helium porosity (%)	Liquid porosity (%)	Gas permeability (mD)	Liquid permeability (mD)	Swi (%)	Lithology	CT scan grade
1	3.797	5.106	134.045	17.97	16.79	1.83	1.288	24.9	Limestone	2
2	3.747	5.021	125.325	16.48	15.13	2.37	1.716	21.3	Limestone	1
3	3.793	5.106	131.326	17.81	16.46	3.19	2.289	21.2	Limestone	2
4	3.798	5.105	132.570	17.95	15.63	4.47	2.125	21	Limestone	1

**Figure 1:**
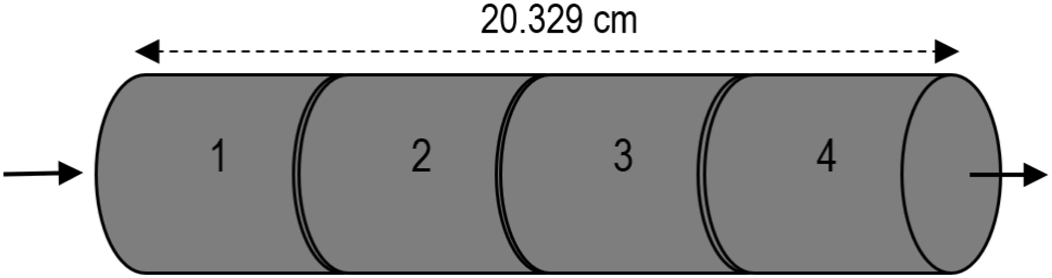
Arrangement of core plugs in the long core.

Dried core plugs were saturated with brine in a vacuum vessel and then pressurized to fully saturate the pore space at 2000 psi for 48 h. The samples were then weighed again and their liquid porosity was calculated. Afterward, the core plugs were placed inside the core holder individually and their liquid permeability was measured by injecting brine with four different rates in room conditions. The samples were then centrifuged with synthetic oil at 11,000 rpm to achieve irreducible water saturation. Next, the wettability of the core plugs was restored through crude oil injection at 60 °C for four days after that the synthetic oil was displaced from pores by injecting decalin as a buffer fluid in a core holder. Figure 1 shows how the long core was assembled from the core plugs, and its average petrophysical properties are listed in Table 5.

**Table 5:** Average petrophysical characteristics of the long core.

Length (cm)	20.329
Diameter (cm)	3.783
Liquid pore volume (cc)	38.005
Bulk volume (cc)	228.554
Liquid permeability (mD)	1.64
Connate water saturation (%)	28
Initial oil saturation (%)	72

#### CO_2_ and flue gas

Commercial-grade of CO_2_ cylinders with a purity of 99.999% were used to inject CO2. A power plant's exhaust, which is the main candidate for the flue gas injection method and located nearby, was sampled. Table 6 provides an illustration of its composition.

**Table 6:** Flue gas composition.

Component	N_2_	CO_2_	O_2_	SO_2_	CO	NO	NO_2_
mol %	82.47	10.38	6.14	0.47	0.13	0.36	0.05

### Core flooding experimental setup and procedure

**Figure 2:**
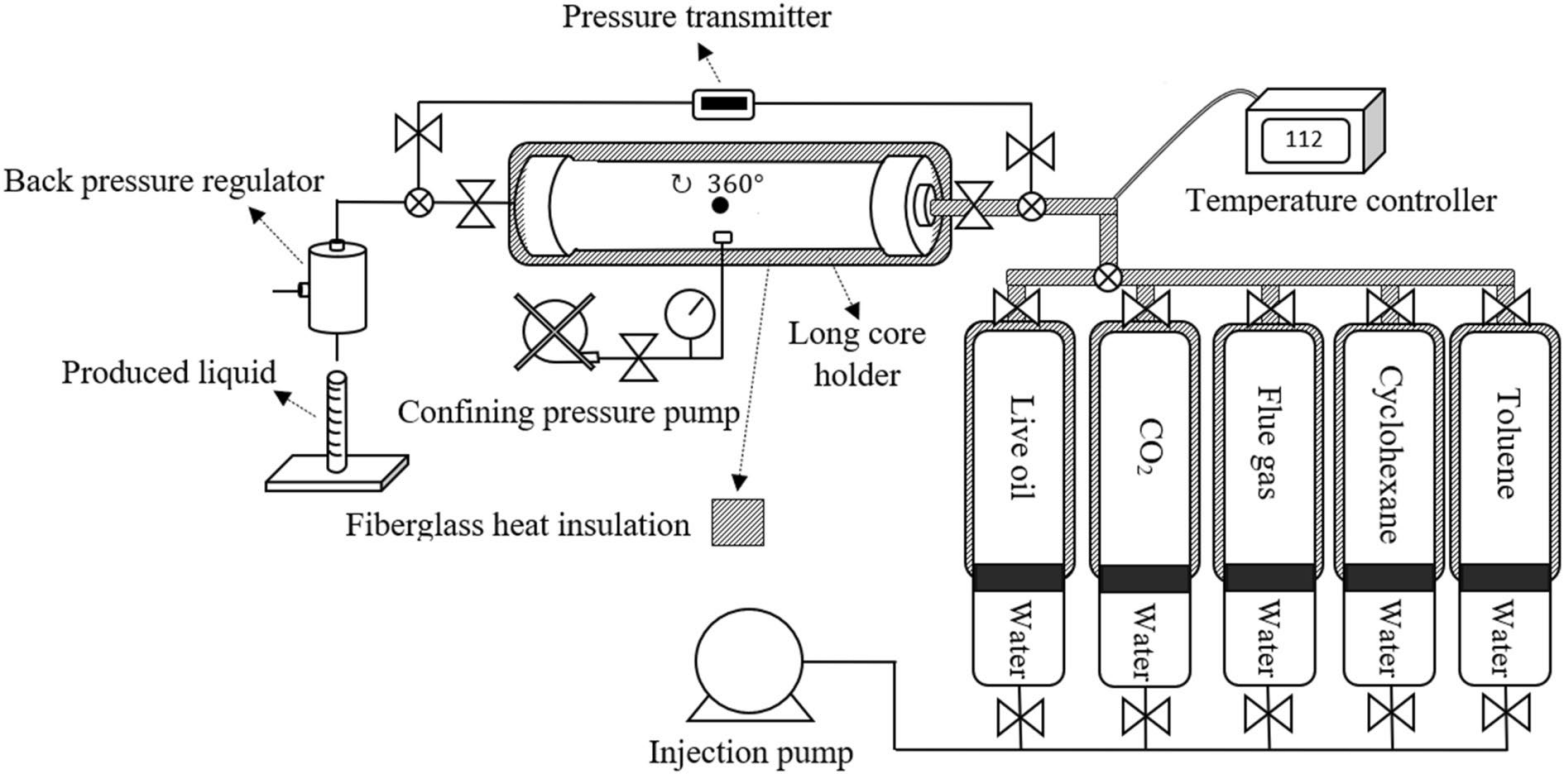
A visual representation of the setup for the experiment.

Figure 2 depicts the core flooding setup, which primary components are as follows. Two automatic positive displacement pumps with the accuracy of 0.001 cc/min, six high-pressure stainless-steel cylinders, a long core holder, and a temperature controller. Water was injected in the annular gap between the sleeve and the core holder with a hand-operated pump to apply the confining pressure to deter the injected fluid from bypassing. In order to adjust the flow direction, the core holder was able to rotate 360°. Rosemount digital pressure transmitter with the accuracy of 0.0001 bar was installed to measure the variation in the amount of pressure exerted on each end of the core holder. High-pressure stainless-steel cylinders containing reservoir brine, live oil sample, CO_2_, cyclohexane, and toluene were connected to the inlet port of the core holder, and a back pressure regulator was installed on its outlet port. The temperature of the core holder, its inlet line, and the cylinders were controlled utilizing a temperature controller at 112 °C. Also, fiberglass heat insulation was installed to prevent any temperature fluctuations.

Cyclohexane does not cause asphaltene to precipitate and cannot dissolve the deposited asphaltene, therefore, it was used to dynamically investigate the formation damage induced by asphaltene deposition via measuring its mobility in the porous media before and after gas injection. First, the four core plugs were placed in the long core holder separately, a confining pressure of 1000 psi, as well as the temperature and the back-pressure, were applied and cyclohexane was injected. As the injection pressure increased higher confining pressure was applied as well to prevent the injecting fluid from bypassing. The confining pressure was maintained 500 psi above the injection pressure during the entire test to minimize the possible mechanical damages to the core plugs.

After that the experimental conditions were stabilized, the cyclohexane mobility in the porous media was calculated via the recorded differential pressure at four different flow rates. In the next stage, the four core plugs were placed in the long core holder to form a long core, and the above-mentioned steps were repeated to measure cyclohexane mobility. Next, to displace the cyclohexane from the pore space, up to 5 times the pore volume, live oil was injected at a low rate of 0.05 cc/min. Then, at a constant rate of 0.05 cc/min, 1.2 pore volume gas (5000 psi, 112 °C) was injected. The recovery factor was calculated by multiplying the volume of the produced oil by the oil formation volume factor (Bo) and dividing the obtained number by the initial oil-in-place volume. To study the formation damage caused by asphaltene precipitation, cyclohexane was then injected until the effluent was transparent and the oil and gas were completely displaced. More effective gas displacement was achieved by shifting the flow direction upward. The injection of cyclohexane was then resumed at four different rates to measure its mobility and it was compared with the prior one to study asphaltene deposition attributed damage.

After measuring cyclohexane mobility in the long core, the core plugs were taken out from the long core holder and reinserted individually. After applying and stabilizing the test conditions, the mobility of cyclohexane in each one of them was measured again, and it was compared with that measured before gas injection. Then, toluene injection started and continued until the output became transparent. The output solution of asphaltene and toluene from each core was collected, passed through a filter paper to eliminate any impurities, finally dried in an oven to measure the weight of the remaining deposited asphaltene for each core plug.

At the end of each set of experiments, the core plugs were washed and dried. Their routine petrophysical characteristics were then re-measure and compared them the initial values to determine any possible mechanical damages.

## Results and discussion

### Recovery factor

The produced oil volume after CO_2_ injection at the room condition is about 14 cc. The Bo value is 1/6369 bbl/STB, therefore the volume of produced oil at reservoir condition is about 24 cc. Since there is 28 cc of live oil in the core at reservoir condition, the recovery factor is about 86%. When flue gas injection is considered, the volume of produced oil at reservoir condition is about 10 cc and the recovery factor is about 36%. Also the Interfacial Tension of gases with reservoir oil was measured experimentally by using the pendent drop method. This result was quite expected due to the large difference in IFT of two systems measured in the test conditions. The large difference between the IFT in the two systems clearly shows that the power of oil sweeping and the amount of oil production in CO_2_ injection is more than that of flue gas.

Due to CO_2_ characteristics, including its high miscibility with oil, which causes lower oil viscosity and lower interfacial tension under suitable conditions of temperature and pressure, CO_2_ gas injection produces more oil than other gases, for this reason it is used in various reservoirs. Due to the mentioned characteristics, the injection of CO_2_ creates a high sweeping efficiency, and also the less viscous fingering phenomenon will occur, which causes the amount of residual oil in the rock to decrease. But flue gas is not used in reservoirs like CO_2_. It usually produces less oil. Flue gas is difficult to dissolve in oil, and for this reason, it does not cause other results of dissolving in oil, such as reducing oil viscosity and interfacial tension, high sweeping efficiency and low viscous fingering similar to CO_2_. In general, the minimum miscibility pressure of flue gas is high compared to other gases^49^.

### Permeability impairment

#### Long core

**Figure 3:**
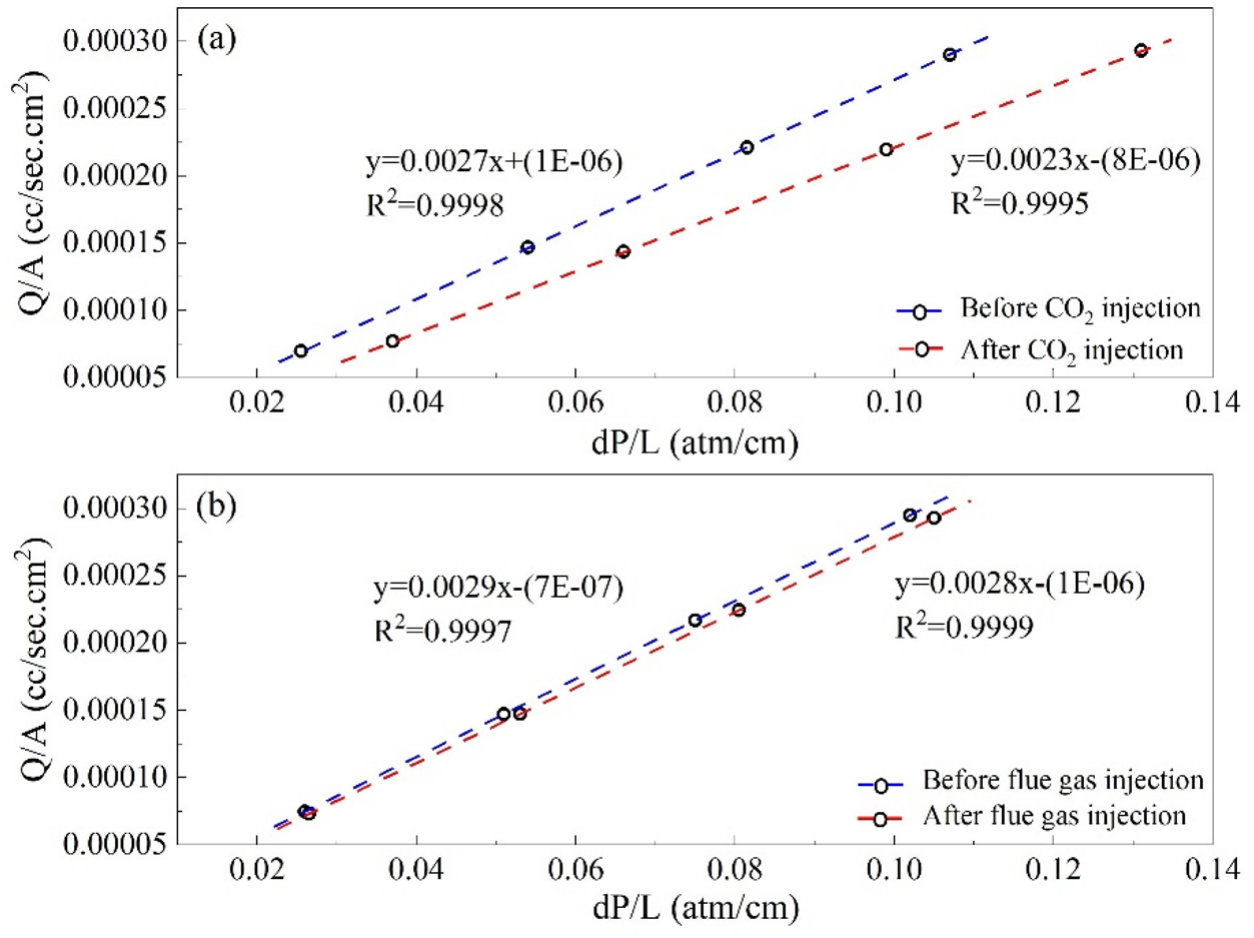
Change in the cyclohexane mobility after gas injection and before live oil injection in (**a**) CO_2_ and (**b**) flue gas injection scenarios.

The mobility of cyclohexane after gas injection to that before live oil injection in both CO_2_ and flue gas injection scenarios in the long core is illustrated in Fig. 3. The residual resistance factor (RRF) represents the ratio of cyclohexane mobility before and after gas injection in the core. In reality, the pores' diameters decrease and some of them close as the amount of asphaltene precipitation in the porous medium rises. In conclusion, the RRF parameter rises in proportion to the amount of asphaltene deposited. After CO_2_ injection, cyclohexane mobility decreases significantly, and the RRF is equal to 1.174 which is more than 1, indicating pore damage and pore throat blockage caused by deposition of asphaltene that occurs while injecting CO_2_. As a result, the permeability of the core has lowered by 14.832%. In the case of flue gas injection, Cyclohexane mobility does not change significantly, and the RRF is closer to 1. The results indicate that the formation damage is minor and much less than that of the CO_2_ injection scenario the permeability of the core has lowered by 4.425% after flue gas injection.

#### Individual investigation of core plugs

**Figure 4:**
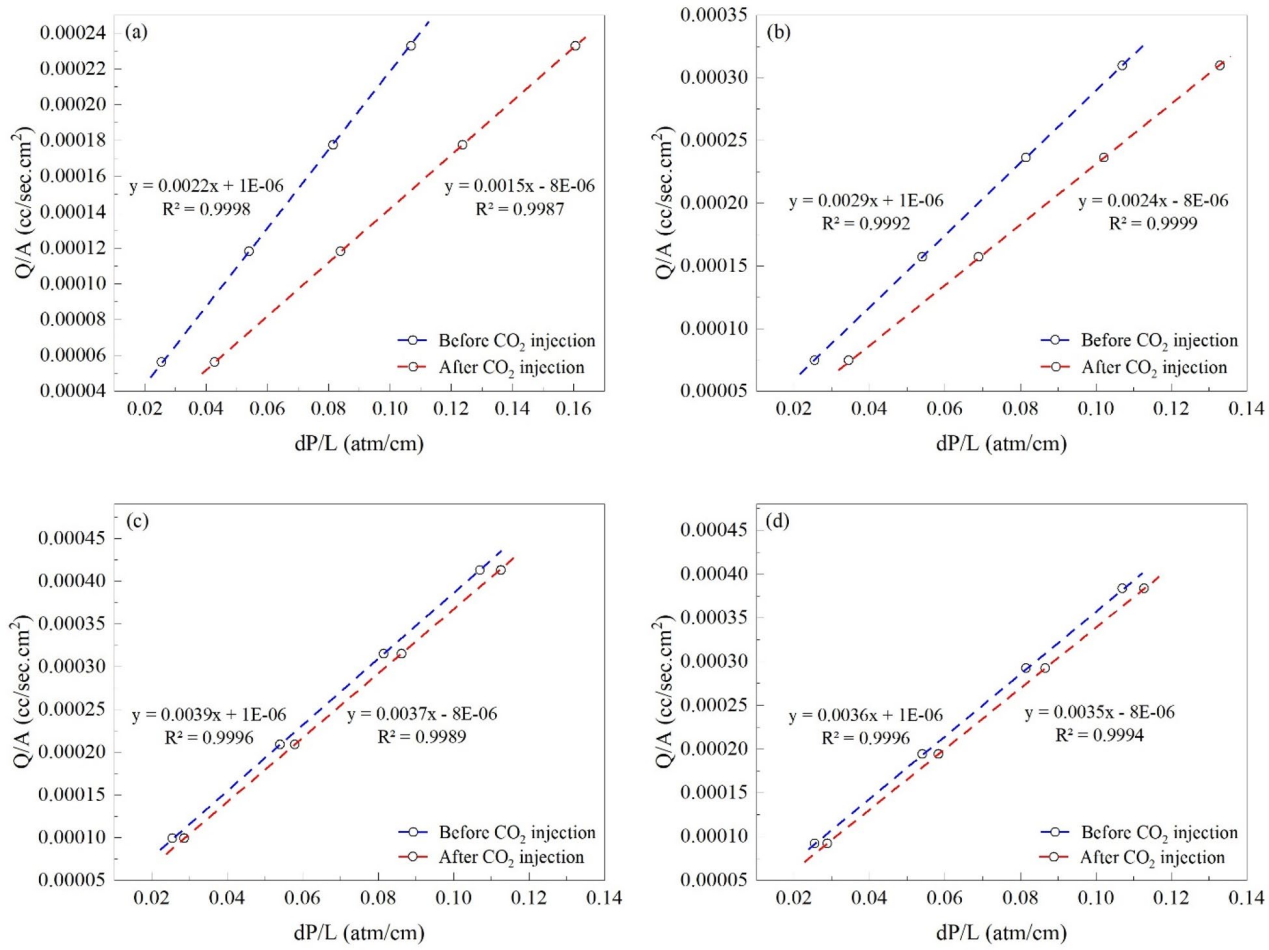
The mobility of cyclohexane before and after CO_2_ injection in the (**a**) first, (**b**) second, (**c**) third, and (**d**) fourth core plug.

**Figure 5:**
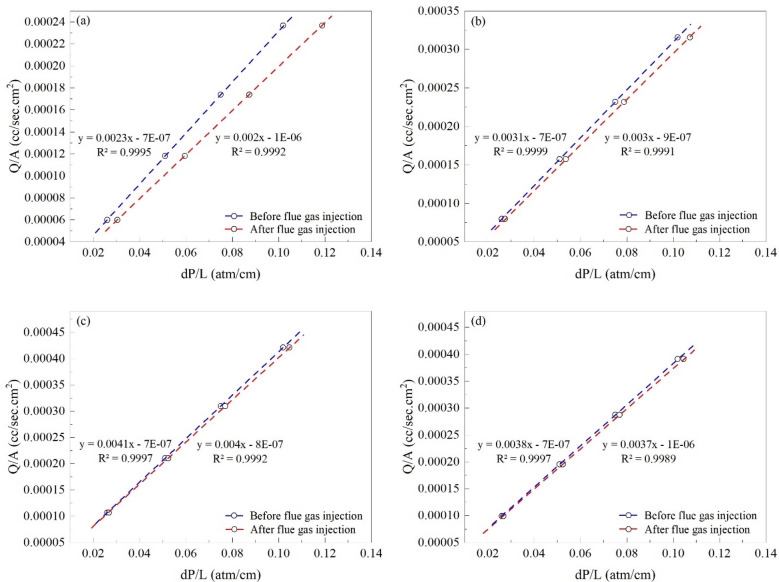
The mobility of cyclohexane before and after flue gas injection in the (**a**) first, (**b**) second, (**c**) third, and (**d**) fourth core plug.

The changes in cyclohexane mobility before and after CO_2_ and flue gas injection for each core plug are illustrated in Figs. 4 and 5, respectively. Cyclohexane mobility is lowered significantly more in the first two core plugs rather than in the last two. As CO_2_ enters the first core plug, the oil in its pore space contacts with the CO_2_ front and deposits its asphaltene content. At the same time, the inlet pressure rises, and oil extraction occurs at the end of the long core. The produced oil at this stage has not come into contact with the CO_2_ front, therefore it is the original oil in place which is produced without depositing its asphaltene content.

Furthermore, since the temperature is constant and the pressure difference between the two ends of the long core is significantly lower than that exists between the reservoir pore space and the production well in a real reservoir, no asphalt deposition occurs in this section. As a result, the quantity of asphalt deposition has reduced as the carbon dioxide front reaches the long core plug end, because the oil in contact with the CO_2_ front has already deposited all of its asphaltene content in the first two core plugs, and there is no more asphaltene to deposit along this path. For the first to last core plugs, the RRF values are 1.466, 1.208, 1.054, and 1.028. Furthermore, the permeability of the first core plug has decreased by 31.818%, while the following three have decreased by 17.24%, 5.128%, and 2.777%, respectively.

Flue gas injection results in a smaller decrease in cyclohexane mobility compared to CO_2_, and except for the first core plug, cyclohexane mobility does not change much. The RRF for the first to the last core plugs is 1.15, 1.033, 1.025, and 1.027, respectively. The permeability has also reduced by 13.04% in the first core plug, and 3.225%, 2.439%, and 2.631% in the remaining three.

To investigate the variations in petrophysical properties caused by mechanical stresses, measurements were conducted on the helium porosity and gas permeability of the samples. The changes are negligible meaning the petrophysical properties are altered only due to asphaltene deposition and not the mechanical stresses.

#### Asphaltene deposition

After toluene evaporation from the container in which the solution of the asphaltene and toluene is poured into through a filter paper, the weight of the residual asphaltene is measured. The first core plug had 78.5 mg of deposited asphaltene, the second one has 39.9 mg, and the third and fourth core plugs each has 23.2 and 19.0 mg of deposited asphaltene. The first core plug, which represents the formation surrounding the injection well, has by far the most deposited asphaltene. Regarding the flue gas, 28.9 mg of asphaltene have been deposited in the first core plug, 19.2 mg in the second one, and 15.7 and 14.3 mg in the remaining two. When compared to CO_2_ injection, the total quantity of deposited asphaltene is noticeably reduced. However, in both scenarios, the pattern of variations in the quantity of deposited asphaltene from the first to the last core plug is nearly identical, with the first one having the most asphaltene deposition.

According to the colloid stability theory, asphaltene exists as a dispersed phase in the oil colloidal system surrounded by resins and aromatics. As long as the equilibrium of this system is not disturbed, asphaltene deposition will not occur, but if the equilibrium is disturbed due to various factors such as changes in temperature, pressure, or oil composition, asphaltene deposition will occur. Gas injection causes the deposition of asphaltene in reservoirs by disrupting the equilibrium state, especially changing the composition of oil. Based on the results of various studies^42,46^, the effect of gas injection on asphaltene deposition is related to the miscibility of oil and gas. In this way, in the miscibility process of oil and gas, there is a change in the composition of oil, which causes the disturbance of the equilibrium and deposition of asphaltene. Usually, CO_2_ gas has a low minimum miscibility pressure (MMP) and flue gas has a high MMP due to the high percentage of nitrogen gas. For this reason, the miscibility possibility of oil and CO_2_ is more than that of flue gas, as a result, more asphaltene deposition occurs in the injection of CO_2_, and the injection of flue gas causes little deposition. On the other hand, since the contact front of oil and gases moves forward, the oil that is in the porous media of plugs 2, 3 and 4 moves towards the outlet of the core holder and exits the long core. In other words, it is produced. This oil has not been in contact with gases. The higher the contact surface between oil and gas, the more the composition of oil will change, and the miscibility possibility of gas and oil will increase. As a result, its equilibrium is disrupted and more asphaltene deposition occurs. Therefore, asphaltene deposition was reduced in the first to the last core plug due to the reduction of contact between oil and gases. Based on the results, it can be seen that the injection of CO_2_ gas produced more oil than the injection of flue gas, despite causing more asphaltene deposition. In other words, other benefits of CO_2_ injection, including reduction of oil viscosity, reduction of interfacial tension and higher miscibility with oil, were able to overcome the negative effect of asphaltene deposition, and it has created a suitable oil recovery factor.

## Conclusion

In the present investigation, the impact of two gas injection scenarios, namely CO_2_ and flue gas, on both oil recovery and formation damage within a lengthy core sample (20.329 cm) under reservoir conditions was systematically examined. In terms of oil recovery, it was determined that CO_2_ gas injection yielded an oil recovery rate over 2.3 times greater than that achieved with flue gas injection. This disparity can be attributed to the lower pressure required for miscibility and, consequently, the higher swelling factor exhibited by CO_2_, which enhances oil recovery. However, with regard to asphaltene deposition and associated formation damage, it was observed that the alteration of oil composition during CO_2_ injection scenarios led to a notable reduction in core permeability, amounting to 14.832%. In contrast, the formation damage resulting from flue gas injection was comparatively minor, causing a permeability reduction of only 4.425%. This suggests that while flue gas injection may yield lower oil recovery, it offers superior flow assurance. But other benefits of CO_2_ injection, including reduction of oil viscosity, reduction of interfacial tension and higher miscibility with oil, were able to overcome the negative effect of asphaltene deposition, and it has created a suitable oil recovery factor.

On the other hand, an investigation into the placement of deposited asphaltene aggregates revealed that among the four core samples, the first two plugs in direct contact with the injected gas exhibited the highest levels of asphaltene deposition and, consequently, the most substantial reduction in permeability. This observation suggests that precipitated asphaltenes were primarily deposited within the proximity of the gas injection point, with limited lateral movement. Furthermore, the movement of the oil front with a low asphaltene content led to oil production within the last two plugs without any asphaltene deposition. In other words, there was minimal interaction between the injected gas and the oil in the final two plugs.

In the case of CO_2_ injection, the permeability reductions in the first and last plugs amounted to 31.81% and 2.77%, respectively, while for flue gas injection, these reductions were measured at 13.04% and 2.63%. Consequently, it can be inferred that when comparing CO_2_ and flue gas injection, particularly in scenarios with a risk of asphaltene deposition, careful consideration must be given to the potential permeability reduction in the vicinity of the injection well.

## Data Availability

The authors declare that, all data generated or analysed during this study are included in this published article.
